# The Impact of Iron Adsorption on the Electronic and Photocatalytic Properties of the Zinc Oxide (0001) Surface: A First-Principles Study

**DOI:** 10.3390/ma11030417

**Published:** 2018-03-12

**Authors:** Jingsi Cheng, Ping Wang, Chao Hua, Yintang Yang, Zhiyong Zhang

**Affiliations:** 1 State Key Laboratory of Integrated Service Networks, School of Telecommunications Engineering, Xidian University, Xi’an 710071, China; jingsicheng@stu.xidian.edu.cn; 2 Institute of Process Engineering, Chinese Academy of Sciences, Beijing 100190, China; huachao@home.ipe.ac.cn; 3 School of Microelectronics, Xidian University, Xi’an 710071, China; ytyang@xidian.edu.cn; 4 College of Electronic and Informational Engineering, Northwestern University, Xi’an 710127, China; zhangzy@nwu.edu.cn

**Keywords:** ZnO (0001) surface, first-principles, iron, optical properties, photocatalytic

## Abstract

The structural stability, electronic structure, and optical properties of an iron-adsorbed ZnO (0001) surface with three high-symmetry adsorption sites are investigated with first-principle calculations on the basis of density functional theory and the Hubbard-U method. It is found that the iron adatom in the H_3_ adsorption site of ZnO (0001) surface has the lowest adsorption energy of −5.665 eV compared with T_4_ and Top sites. For the Top site, compared with the pristine ZnO (0001) surface, the absorption peak located at 1.17 eV has a red shift, and the elevation of the absorption coefficient is more pronounced in the visible-light region, because the Fe-related levels are introduced in the forbidden band and near the Fermi level. The electrostatic potential computation reveals that the work function of the ZnO (0001) surface is significantly decreased from 2.340 to 1.768 eV when iron is adsorbed on the Top site. Furthermore, the degradation mechanism based on the band structure is analyzed. It can be concluded that the adsorption of iron will promote the separation of photoinduced carriers, thus improving the photocatalytic activity of ZnO (0001) surface. Our study benefits research on the photocatalytic activity of ZnO and the utilization rate of solar energy.

## 1. Introduction

In recent years, with increasingly serious environmental pollution and energy shortage, semiconductor-based photocatalysts have aroused great interest. Normally, semiconductor photocatalytic technology is based on energy band theory. When the incident photon energy is larger than the band gap energy (*E*_g_) of the semiconductor, electrons (e^−^) will be excited and promoted from the valence band (VB) to the conduction band (CB), leaving an equal number of holes (h^+^) behind [1]. Then, the electrons and holes will combine with O_2_ and H_2_O, forming ${\cdot O}_{2}^{-}$ and ·OH to react with the organic pollutants as they spread to the surface of the semiconductor. TiO_2_ [2,3,4], CdS [5,6], ZnO [7,8], and MoO_3_ [9,10] are good photocatalysts and, under a certain intensity of light irradiation, are used in wastewater treatment to degrade organic pollutants. Zinc oxide (ZnO), a potential semiconductor photocatalyst, has a low cost, is chemically stable, is non-toxic, and has a high photocatalytic effect on organic pollutants because of its large excitation binding energy (*E_b_* = 60 meV) [11,12]. However, its photocatalytic activity is restricted to the ultraviolet (UV) region (wavelength below 400 nm) of the solar spectrum by its wide band gap (3.37 eV) at room temperature [13]. The increase of absorption coefficient in the visible light region can improve the utilization rate of sunlight and the photocatalytic spectrum range of ZnO. Some experimental and theoretical works have been undertaken to enhance the absorption coefficient of ZnO under visible light irradiation with foreign element-doping and the surface modification of, for example, semiconductor composite and noble metal composite structures [14,15,16,17,18]. Yu et al. [15] revealed that the light absorption coefficient of ZnO in the visible light region was greatly enhanced by N- and C-doping through first-principle calculations. Li et al. [16] observed that doped Co^2+^ ions can prevent the recombination of photoinduced electron–hole pairs and decrease the optical band gap, whereas the ZnO/CuS composite heterostructure can increase the visible light absorption coefficient and enhance the separation of photoexcited charge carriers by conducting methyl orange (MO) photodegradation experiments. Cheng et al. [18] showed that the absorption edge of Ag/ZnO core-shell nanoparticles had a red shift and a larger light absorption coefficient compared with that of the bulk ZnO by the first-principle computations using the Vienna ab initio simulation package (VASP).

The morphology of the ZnO surface will induce a better photocatalytic activity because a higher surface area possesses several active sites for the interaction with pollutants [19,20]. As is known, the morphology of ZnO is dominated by four main low-Miller-index surfaces: polar (0001) and ($000\bar{1}$) surfaces as well as non-polar ($10\bar{1}0$) and ($11\bar{2}0$) surfaces. Zhang et al. [21] investigated these four surfaces by first-principle calculations with VASP and confirmed that (0001)-Zn surface had the strongest absorption in the near UV region range among these four surfaces and a remarkable red-shift phenomenon of the absorption edge compared with the bulk ZnO, indicating that (0001)-Zn surface had the highest photocatalytic activity with low photon energy. In recent years, the adsorption of the Cu, Co, Ag, Au, Mg, B, and Si atoms on the ZnO (0001) surface has been investigated [22,23,24,25,26,27]. Warschkow et al. [22] studied the stability of Cu-exposed ZnO (0001) surface structures in an oxygen environment using an ab initio atomistic thermodynamics method based on density functional theory (DFT), confirming that two structures with a ($\sqrt{3}\times \sqrt{3}$)R30° unit cell were prominently stable at intermediate oxygen and copper chemical potentials. Lyle et al. [23] studied the Cu/ZnO (0001) surface with methanol via DFT calculations and found that more favorable energy was identified at higher coverage (1/4 ML versus 1/16 ML). Yang et al. [26] investigated Ag- and Au-adsorbed ZnO (0001) surfaces by first-principle calculations. And they found that Ag and Au atoms preferred to be adsorbed on the H_3_ sites of the surface, and Ag- and Au-adsorbed ZnO (0001) surfaces exhibited metal characteristics. Furthermore, Zhang et al. [27] investigated Si-adsorbed ZnO (0001) surface by first-principle calculations and observed that H_3_ sites played a critical role in the red shift of absorption edge. Iron (Fe), as an important transition metal element, also plays a key role in human life because it is abundant on earth (4.75%), and iron-doped ZnO has shown better photocatalytic performance than that of intrinsic ZnO, because an iron dopant generates a deep trap level at *E*_v_ + 1.01 eV in the band gap of ZnO [28]. However, the impact of iron atom adsorption on ZnO (0001) surface, to the best of our knowledge, has not been reported.

This paper presents a theoretical investigation of the effect of iron atom adsorption on the structural stability, electronic structure, and optical properties of a ZnO (0001) surface using first-principle computations. The surface work function of three adsorption sites on a ZnO (0001) surface is studied by electrostatic potential calculations. The photocatalytic performance of an iron-adsorbed ZnO (0001) surface is further discussed.

## 2. Calculation Models and Methods

All geometric structures in this work are calculated by the Cambridge Serial Total Energy Package (CASTEP) module of the Materials studio software on the basis of DFT [29,30]. The exchange-correlation energies are described by the generalized gradient approximation (GGA) with Perdew–Burke–Ernzerh (PBE) [31,32], and a plane-wave cutoff energy of 400 eV is adopted. The valence-electron configurations are Zn 3*d*^10^4*s*^2^, O 2*s*^2^2*p*^4^, and Fe 3*d*^6^4*s*^2^. All systems are calculated in reciprocal space, and the Monkhorst–Pack grid of (4 × 4 × 1) k-points is used. For the geometric structure relaxation, the energy charge and maximum tolerances of the force, stress, and displacement are set to 1 × 10^−5^ eV/atom, 0.03 eV/Å, 0.05 GPa, and 0.001 Å, respectively.

**Figure 1:**
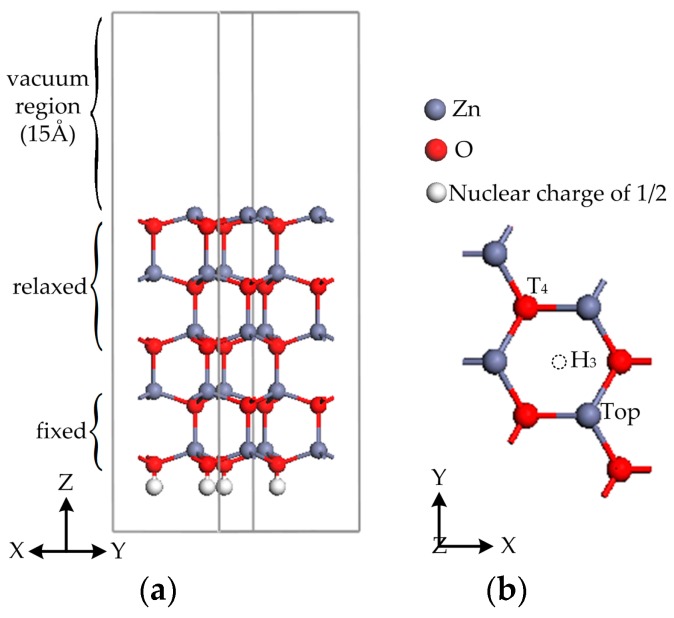
The side view (**a**) and top view (**b**) models of the pristine (2 × 2) ZnO (0001) surface and three adsorption sites.

On the basis of the optimized wurtzite ZnO unit cell, a cross-sectional plane of ZnO perpendicular to (0001) could be a Zn-rich (Zn-terminated) or O-rich (O-terminated) surface because bulk ZnO consists of Zn and O slabs alternatively along the (0001) direction [33]. As is shown in Figure 1, the Zn-terminated (0001) polar surface is constructed by a (2 × 2) surface unit cell slab geometry of periodically repeated supercells including five Zn–O bilayers (10 atomic layers). The top three Zn–O bilayers and the adatom layer are allowed to relax, while the two bottom Zn–O bilayers are fixed to mimic the bulk structure. To eliminate the interaction between the two adjacent slabs, the surface supercells are separated from each other by the vacuum region, which is set to 15 Å along the *z*-axis. The pseudo-hydrogens with a nuclear charge of 1/2 for a Zn-terminated surface are used to prevent the unphysical charge transfer between the top and bottom slabs. For an iron-adsorbed ZnO (0001) surface, three high-symmetry adsorption sites are considered: the H_3_ site with no atom beneath, the T_4_ site above a surface atom, and the Top site located at just above a surface atom.

As is known, the adsorption energy per iron atom is calculated by the following formula [34]:

(1) $$E_{a}=E_{tot}-E_{slab}-\mu_{Fe}$$

where *E_a_* is the adsorption energy, *E_tot_* is the total energy of the surface system after iron adsorption, *E_slab_* is the energy of the clean ZnO (0001) surface, and $\mu_{Fe}$ is the chemical potential of a single iron atom.

The surface work function *W_f_* is also a very significant property in the surface science, which is defined as the minimum energy required to remove an electron from the bulk of a material through a surface to a point in vacuum immediately outside the surface. In the calculation of the work function, the surface is assumed to be in its ground state both before and after removal of the electron [35]. In this work, under the conditions of a 0 K temperature and a perfect vacuum, the work function can be obtained as follows [36,37]:

(2) $$W_{f}=E_{vac}-E_{f}$$

where *E_vac_* is the converged electrostatic potential energy over the supercell slab surface, and the Fermi level energy (*E_f_*) is related to the highest occupied electronic state of the system.

To investigate the effect of iron adsorption on the photocatalytic properties of the ZnO (0001) surface, the optical properties are considered. Here, the calculated band gap of the bulk ZnO, based on the GGA method, was 0.74 eV, which is smaller than the experimental value of 3.37 eV [12]. As is known, the GGA method underestimates the band gap of transition metal oxides with a strong exchange correlation effect. Thus, spin-polarized GGA+U electronic structure calculations are adopted to accurately describe the electronic structure [38], and the Hubbard parameters *U_d_* for Zn 3*d* states and *U_p_* for O 2*p* states are considered to be 10 and 7 eV [39,40], respectively. The calculated band gap (3.296 eV) of the bulk ZnO is in good agreement with the experimental value (3.37 eV). Additionally, the *U_d_* = 2.5 eV is added to Fe 3*d* orbits in this work. Moreover, a dense 6 × 6 × 1 k-point mesh for the Brillouin zone is adopted in our optical performance calculation, and the optical properties are studied by the dielectric function of the frequency, which consists of the imaginary part *ε_r_* and the real part *ε_i_*:

(3) $$\varepsilon \left(\omega \right)=\varepsilon_{r}\left(\omega \right)+i\varepsilon_{i}\left(\omega \right).$$

The imaginary part of the dielectric function *ε_i_* (*ω*) is determined by a summation over empty band states [41]:

(4) $$\varepsilon_{i}\left(\omega \right)=\frac{2\pi e^{2}}{\Omega \varepsilon_{0}}\underset{k,v,c}{\sum }\delta \left(E_{k}^{c}-E_{k}^{v}-\hbar \omega \right){\left|\langle \psi_{k}^{c}\left|u\cdot r\right|\psi_{k}^{v}\rangle \right|}^{2}$$

where *ω* is a given angular frequency of incident photons, *u* is the vector defining the polarization of the incident electric field, Ω is the unit cell volume; *k* is the reciprocal lattice vector, *v* and *c* denote the conduction and valence band states, respectively, and $\psi_{k}^{c}$ and $\psi_{k}^{v}$ are the wave functions of the conduction and valence bands, respectively. The real part ε*_r_* (*ω*) can be calculated from *ε_i_* (*ω*) using the Kramers–Kronig relations [42] as follows:

(5) $$\varepsilon_{r}\left(\omega \right)=1+\frac{2}{\pi }\int_{0}^{\infty }\frac{\omega^{\prime }\varepsilon_{i}\left(\omega^{\prime }\right)}{\left(\omega^{\prime }{}^{2}-\omega^{2}\right)}d\omega^{\prime }.$$

Then, the other optical constants can be obtained from *ε_r_* (*ω*) and *ε_i_* (*ω*), such as the absorption coefficient *α* (*ω*), which can be expressed as [43]:

(6) $$\alpha \left(\omega \right)=\sqrt{2}\omega {\left[\sqrt{\varepsilon_{r}^{2}\left(\omega \right)+\varepsilon_{i}^{2}\left(\omega \right)}-\varepsilon_{r}\left(\omega \right)\right]}^{1/2}.$$

## 3. Results and Discussion

### 3.1. Geometries and Structural Stability

**Figure 2:**
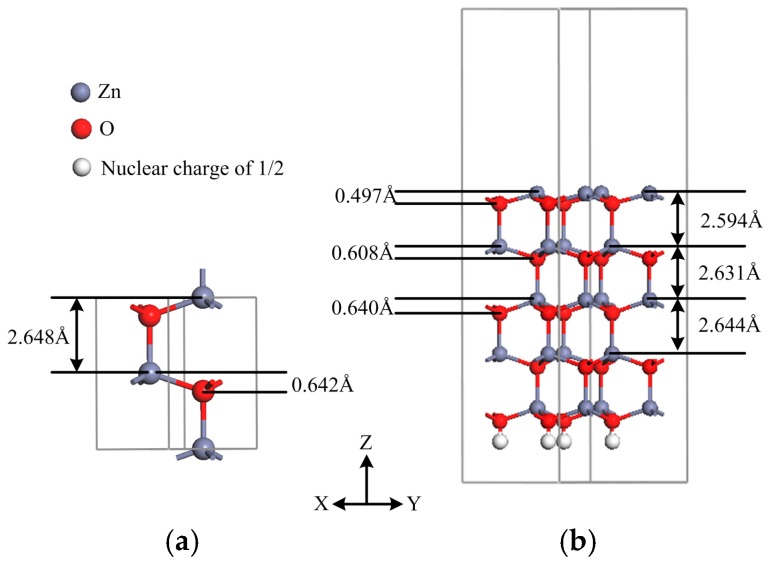
Schematic models of (**a**) the bulk wurtzite ZnO unit cell and (**b**) the pristine (2 × 2) (0001) surface.

Table 1 summarizes the obtained lattice constants of bulk wurtzite ZnO unit cell, which matches well with previous investigation results, thereby confirming the correctness of our calculation. Figure 2 shows the geometric structures of the bulk ZnO and the pristine ZnO (0001) surface. Figure 2a,b show that both the spacings of Zn and O atoms in the same Zn–O bilayer and the distances between the zinc atomic planes of the relaxed Zn–O bilayers of ZnO (0001) surface are all smaller than those of the bulk ZnO, respectively, which is due to the compression at the relaxed layers, and this phenomenon is confirmed by Ag- or Au-adsorbed ZnO (0001) surfaces [26]. Meanwhile, the smaller the spacing and distance of the outer bilayers are, the weaker the lattice constraint is at the relaxed surface.

**Figure 3:**
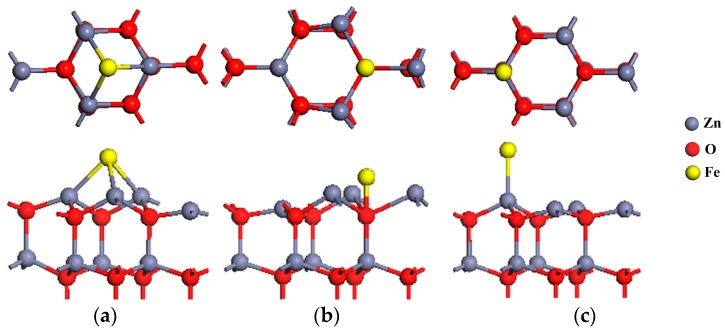
The side and top view of the optimized structures of iron atom adsorbed on (**a**) the H_3_ site; (**b**) the T_4_ site; (**c**) the Top site of ZnO (0001) surface.

Figure 3 shows the geometric structures of ZnO (0001) surface with three high symmetry iron adsorption sites. As is observed, the iron atoms have a significant impact on the adjacent zinc and oxygen atoms. Table 2 lists the total energy, bond length, and adsorption energy of the iron adatoms for these three sites. It can be found that, after the geometrical optimization, when the iron atoms are adsorbed on H_3_ and T_4_ sites, each iron adatom will have three neighboring zinc atoms within the distances of 2.482 and 2.318 Å, respectively. The iron atom adsorbed at T_4_ will form a bond with the oxygen atom just below the site, with a bond length of 1.703 Å. At the Top site, the iron atom will attract one zinc atom from the surface within a distance of 2.339 Å. Based on the surface adsorption energy calculation, all of the adsorption sites can be easily adsorbed on the ZnO (0001) surface. This is because the surface adsorption energy of these three sites is negative and small, so the adhesion of the iron atom on the ZnO (0001) surface is easier and the iron-adsorbed ZnO surface will be quite stable. Specifically, the H_3_ site has the lowest adsorption energy of −5.665 eV, so the adsorption of iron adatom on the H_3_ site is the most favorable compared with the other two sites, and this result is consistent with the former reports in [33,46].

### 3.2. Electronic Structure

**Figure 4:**
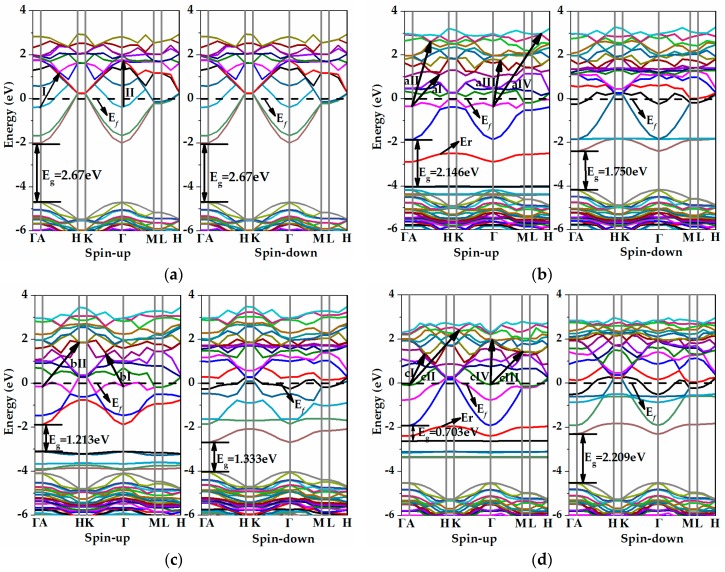
The band structures of (**a**) pristine ZnO (0001) and iron atom adsorbed on (**b**) the H_3_ site, (**c**) the T_4_ site, and (**d**) the Top site of the ZnO (0001) surface via the GGA+U method.

Figure 4 presents the computed spin-polarized band structures of the pristine and iron-adsorbed ZnO (0001) surfaces via the GGA+U approach. The Fermi level is set as zero in the energy level. As can be seen in Figure 4a, the calculated band structure of the pristine ZnO (0001) surface exhibits a direct band gap because the conduction band minimum (CBM) and the valence band maximum (VBM) are situated at the same Γ point of the Brillouin zone. The achieved band gap of the pristine ZnO (0001) surface is 2.67 eV, which is smaller than that of the intrinsic bulk ZnO counterpart (about 3.296 eV) due to the occurrence of the surface state band in the bulk band gap and the electric field in the slab induced by band bending [21,47,48]. In addition, the Fermi level shifts the conduction band (CB) upward, and the quantum states near the CBM are then occupied by electrons, indicating that the ZnO (0001) surface is an n-type degenerate semiconductor with metallic characteristics. This is mainly because the shallow donor states are created in the bottom of the CB, inducing an increase in the carrier concentration. Figure 4b–d demonstrate the band structures of the iron-adsorbed ZnO (0001) surfaces. It can be observed that they are similar to that of the pristine ZnO (0001) surface. The band structure shows a direct gap at the Γ point when the iron atom is adsorbed on the ZnO (0001) surface, and the Fermi level of both spin-up and spin-down channels also lie in the CB. It is worth mentioning that the band gaps in the spin-up and spin-down channels of all adsorbed ZnO surface systems are smaller than those of the pristine ZnO (0001) surface (2.67 eV). Specifically, in Figure 4b,d showing the band structures in the spin-up channel of the H_3_ and Top configurations, the impurity levels (*E_r_*) located at −2.91 and −2.38 eV are created in the forbidden band at the Γ point, respectively, and the *E_r_* may become effective recombination centers. Figure 4c shows the band structure of T_4_ configuration. It is found that the iron adatom does not produce an obvious *E_r_*, whereas the band gaps in both spin channels significantly decrease to 1.213 and 1.333 eV, respectively. Notably, there are several Fe-related levels near the Fermi level of these two spin channels and the upper part of the VB in the spin-up channel. Furthermore, under the irradiation of the solar spectrum, because of the impact of the Fe-related level on the vicinity to the Fermi level, the Fermi level will function as a “springboard” for electrons to jump into the CB, which may lead to a stronger optical absorption coefficient in the visible light region [15].

**Figure 5:**
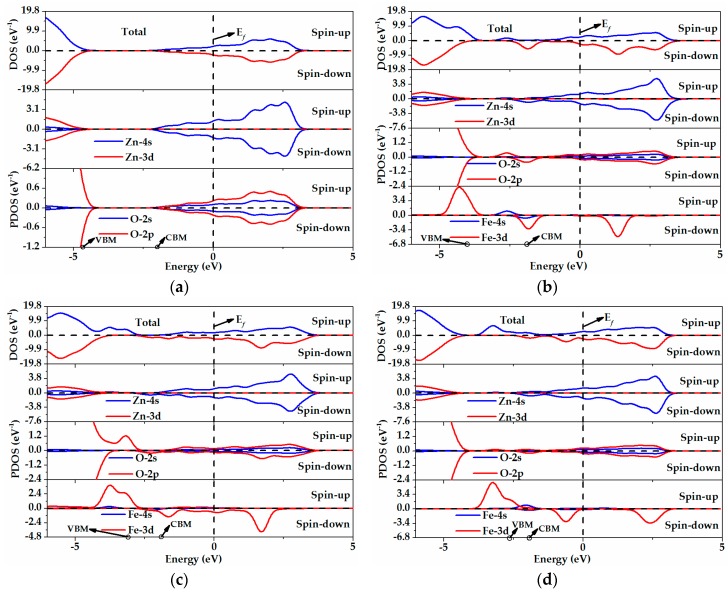
The total density of state (TDOS) and the projected density of state (PDOS) of (**a**) the pristine ZnO (0001) and the iron atom adsorbed on (**b**) the H_3_ site, (**c**) the T_4_ site, and (**d**) the Top site of ZnO (0001) surface via the GGA+U method. The positions of the conduction band minimum (CBM) and the valence band maximum (VBM) are also denoted.

The calculated total density of state (TDOS) and the projected density of state (PDOS) of these two spin channels for the pristine and iron-adsorbed ZnO (0001) surfaces are presented in Figure 5, respectively. As is shown in Figure 5a, the CB of the pristine ZnO (0001) surface mainly consists of Zn-4*s* and O-2*p* states, and the VB is mainly composed of Zn-3*d* and O-2*p* states. In addition, the spin-up and spin-down components of the TDOS and PDOS are symmetrical, implying that the pristine ZnO (0001) surface is nonmagnetic, whereas the iron-adsorbed ZnO (0001) surfaces, based on Figure 5b–d, are magnetic due to the fact that the iron element belongs to the transition metal element and that the TDOS of these two spin channels near the Fermi level are asymmetrical. Compared with the pristine ZnO (0001) surface in Figure 5a, the Fe-3*d* states mainly have contribution to the CB in the spin-down channel near the Fermi level and the upper part of the VB in the two spin channels of all the iron-adsorbed ZnO surfaces. Moreover, in the CB with a range from 1 to 4 eV, all the Fe-3*d* states exhibit strong localization, making the CB of iron-adsorbed ZnO (0001) surface more intensive. However, in the upper part of the VB with a range from −5 to −3 eV, there is strong hybridization between the Fe-3*d* and O-2*p* states, implying the strong interaction between iron and oxygen atoms. Moreover, from Figure 5b,d, it can be seen that Fe-4*s* states exhibit localization near the −2.72 and −2.15 eV, respectively, which matches with the results in Figure 4b,d. In particular, it should be noted that, in Figure 5c, there is a stronger interaction between Fe-3*d* and O-2*p* states in the upper part of the VB of the T_4_ site compared with the H_3_ and Top configurations, which further confirms the bond between iron adatom and the surface oxygen atom of the first bilayer in Table 2.

**Figure 6:**
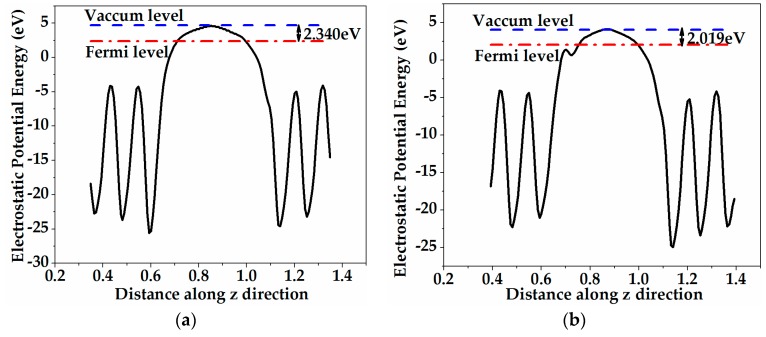

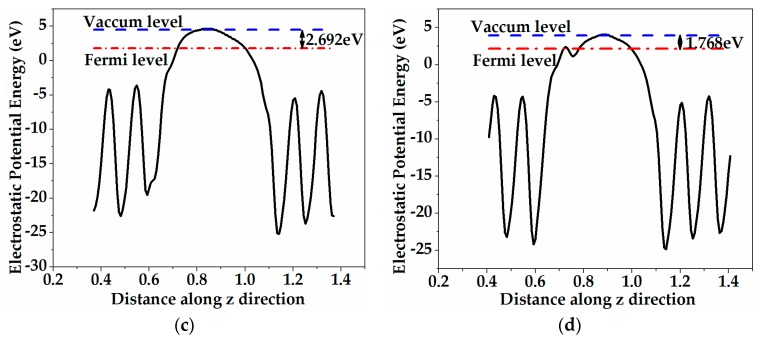
Calculated electrostatic potential of the *z* direction for (**a**) the pristine ZnO (0001) surface and the iron atom adsorbed on (**b**) the H_3_ site, (**c**) the T_4_ site, and (**d**) the Top site of the ZnO (0001) surface.

Figure 6 presents the calculated electrostatic potential of the pristine and iron-adsorbed ZnO (0001) surface systems. The adsorption of iron atom on the ZnO (0001) surface will affect the surface work function that can be achieved by averaging the electrostatic potential along the *z*-axis [35]. Figure 6a gives the electrostatic potential of the pristine ZnO (0001) surface, which tends to be flat in the vacuum region because of the introduction of pseudo-hydrogens, and this result is consistent with previous work [36]. In Figure 6b–d, iron atom adsorbed on the T_4_ site will increase the work function of the ZnO (0001) surface, which is the opposite of the scenarios of the H_3_ and Top adsorption sites. Moreover, compared with the pristine ZnO (0001) surface, the work function of the Top configuration decreases to 1.768 eV and declines most among the three sites. According to Qiao et al. [49,50], the surface work function is sensitive to the charge transfer and surface state concentration. Thus, the variation of the surface work function may be ascribed to the fact that iron adatom is bonded to the oxygen atom in the topmost layer when iron atom is adsorbed on the T_4_ site, and iron adatom will interact with zinc atoms when iron atom is adsorbed on the H_3_ and Top sites. This may lead to the changes of chemical properties and surface states in the (0001) surface. Accordingly, the photocatalytic reaction rate will be promoted.

### 3.3. Optical Properties

**Figure 7:**
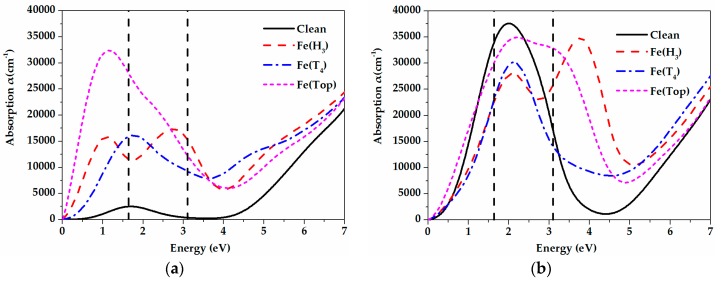
The calculated optical absorption spectra of the pristine and iron-adsorbed ZnO (0001) surfaces along the (**a**) (100) and (**b**) (001) directions. Vertical dash lines represent the energy range of visible light spectrum (i.e., 1.64–3.10 eV correspond to 760–400 nm).

Figure 7 plots the calculated spectra of absorption coefficient (α) for the pristine and iron-adsorbed ZnO (0001) surfaces. The incident radiation has linear polarization along the (100) and (001) directions. As can be seen, compared with the pristine ZnO (0001) surface, the absorption coefficient of the iron-adsorbed ZnO (0001) surface is steeply increased in the visible and infrared (IR) regions along the (100) direction and enhanced in the near-ultraviolet light region along the (001) direction. Meanwhile, the iron atom adsorbed on the Top adsorption site has the highest adsorption coefficient in the region from 0 to 2.6 eV along the (100) direction, and H_3_ configuration has the highest adsorption coefficient in the range from 3.5 to 5.2 eV along the (001) direction. Such enhanced visible light absorption in the iron-adsorbed ZnO (0001) surface may be attributed to the narrow band gaps and the Fe-related levels near the Fermi level. For the scenario of the iron atom adsorbed on the H_3_ and Top sites, as shown in Figure 4b,d, there are obvious Fe-related levels located in the forbidden band with smaller energy gaps of 2.146 and 0.703 eV, which may induce more electronic transitions and thus enhance the optical absorption coefficient. However, for the iron atom adsorbed on the T_4_ site in Figure 4c, the introduced Fe-related levels at the upper part of the VB in the spin-up channel make the band gap smaller than that of the pristine ZnO (0001) surface, therefore improving the absorption coefficient in the visible light region. Table 3 shows the relationship between the transition process (please refer to Figure 4) and the position of adsorption peaks. For the n-type degenerate semiconductors, the Fermi levels will enter into the CB, and the quantum states between the CBM and Fermi level will be occupied by electrons [51]. Thus, the absorption peak is mainly affected by the band gap and the intraband transition of hot electrons. It can be found in Table 3 that the prominent absorption peak in the low energy region of the pristine ZnO (0001) surface is located at 1.72 eV along the (100) direction, which coincides well with data (1.76 eV) in [27]. Compared with it, the absorption peak in the lower-energy region of the H_3_ site is observed at 1.12 eV with a clear red shift. The absorption peak of the T_4_ site is also located at 1.72 eV, whereas the light adsorption is elevated in both the visible light and the near-ultraviolet light regions. Moreover, at the Top site, the absorption peak shifts toward the lower-energy region, but the absorption intensity is strengthened significantly. This is because the compounds adsorbed by the iron atom on the ZnO (0001) surface are all n-type degenerate materials and the absorption peaks are from the intraband transitions of hot electrons.

### 3.4. Photocatalytic Performance

**Figure 8:**
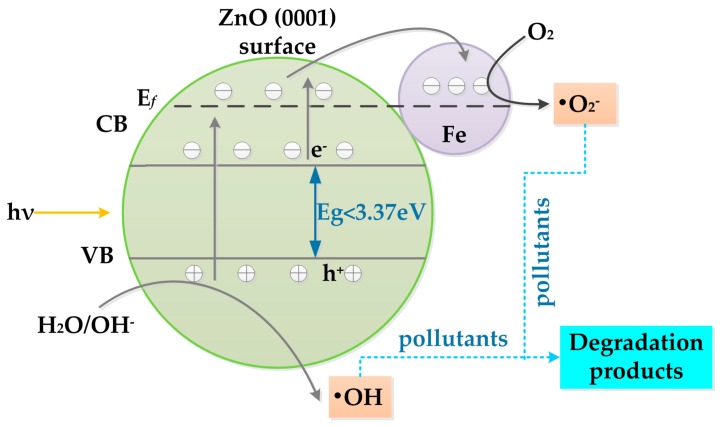
Schematic explanation of the transfer of electron–hole pairs in iron-adsorbed ZnO (0001) surface leading to the degradation of pollutants under sunlight irradiation.

Figure 8 presents the possible photocatalytic reaction mechanism of iron-adsorbed ZnO (0001) surface under visible light irradiation. As can be seen, when the iron-adsorbed ZnO (0001) surface is confronted with solar light irradiation, the photoinduced electrons transfer from the ZnO (0001) surface to the iron atoms. At the same time, the photogenerated holes remain on the surface and the photogenerated electron–hole pairs are separated with a recombination reduction [1], thus enhancing the photocatalytic activity and the degradation rate of the iron-adsorbed ZnO (0001) surface.

During the decomposition process, the photoexcited electrons will react with O_2_ forming superoxide radical anions (·O_2_^−^) and the photoinduced holes will react with H_2_O or OH^−^ to produce hydroxyl radicals (·OH), which can be utilized as species for the degradation of organic pollutants by oxidation [52]. According to the formation process of e^−^/h^+^ pairs, as shown in Figure 8, the possible mechanism of the iron-adsorbed ZnO (0001) surface photocatalyst for the degradation of pollutants can be proposed as follows:

Fe-ZnO (0001) surface + hν → e^−^ + h^+^ (7)

H_2_O/OH^−^ + h^+^ → ·OH (8)

(9) $$O_{2}+e^{-}\to \cdot O_{2}^{-}/\cdot 2O^{-}$$

(10) $$h^{+}+{\cdot O}_{2}^{-}/\cdot 2O^{-}+\cdot \mathrm{OH}+\mathrm{pollutant}\to CO_{2}+H_{2}O$$

From the above photocatalytic process, it can be found that the photocatalytic activity of iron-adsorbed ZnO (0001) surface will be higher than that of the pristine ZnO (0001) surface. This is mainly because the adsorption of iron atom will promote the separation between the electrons and holes, thus prolonging the charge carrier lifetime. Subsequently, more carriers separated will move to the ZnO (0001) surface and participate in the redox reactions under irradiation with visible light. Finally, the organic pollutants are decomposed to the non-toxic CO_2_ and H_2_O.

## 4. Conclusions

The impact of iron adsorption on the structural stability, electronic structure, and optical properties of the pristine and iron-doped ZnO (0001) surface were studied via first-principle computations with the GGA+U method. The results show that the spacings between Zn and O atoms in the same Zn–O bilayer and the distances between the zinc atomic planes of different relaxed Zn–O bilayers of ZnO (0001) surface are shrunk. Iron atoms prefer to be adsorbed on the H_3_ site of ZnO (0001) surface with a low adsorption energy of about −5.665 eV. The band gap of the iron-adsorbed ZnO (0001) surface is lower than that of the pristine ZnO (0001) surface. Particularly, the Fe-related levels are located at the forbidden band and near the Fermi level for the case of iron adsorption on the H_3_ and Top sites, whereas, for the T_4_ site, the Fe-related levels are located at the upper part of the VB and near the Fermi level, inducing more electronic transitions and a significant improvement of the optical absorption coefficient in the visible light energy region. Furthermore, besides the T_4_ site, the work function of the H_3_ and Top sites is also decreased. This work can provide reference for the investigation of the photocatalytic properties of the ZnO (0001) polar surface.

## Figures and Tables

**Table 1:** Calculated and experimental values of the structural parameters for bulk ZnO unit cell. a, b, and c are the lattice parameters.

Lattice Constants	Present Work	Previous Computation ^i^	Experiment ^ii^
a (Å)	3.2820	3.2935	3.250
b (Å)	3.2820	3.2935	3.250
c (Å)	5.2951	5.2877	5.201
c/a	1.6134	1.6050	1.600

^i^ Ref. [44]; ^ii^ Ref. [45].

**Table 2:** The total energy, bond length, and adsorption energy (eV) of the iron-adsorbed ZnO (0001) surface.

Fe-Adsorbed Site	*E_total_* (eV)	Bond Length (Å)		*E_a_* (eV)
		Fe-O	Fe-Zn	
H_3_	−43,845.903	-	2.482	−5.665
T_4_	−43,844.674	1.703	2.318	−4.436
Top	−43,845.403	-	2.339	−5.165
Clean surface	−42,984.348	-	-	-

**Table 3:** The position of adsorption peaks of the iron-adsorbed ZnO (0001) surface along the (100) and (001) directions and the corresponding transition process.

Direction	Clean Surface		Fe(H_3_)		Fe(T_4_)		Fe(Top)	
	Position (eV)	Process	Position (eV)	Process	Position (eV)	Process	Position (eV)	Process
(100)	1.72	I	1.12	aI	1.72	bI	1.17	cI
			2.75	aII			2.30	cII
(001)	1.92	II	2.11	aIII	2.12	bII	2.28	cIII
			3.77	aIV			2.96	cIV
